# Recurrent Phosphoglyceride Crystal Deposition Disease in the Mandible Mimicked a Malignant Tumor: Insights from a Clinical Case Report and Literature Review

**DOI:** 10.3390/diagnostics16040567

**Published:** 2026-02-13

**Authors:** Jumpei Shirakawa, Motoo Ito, Takuya Matsuzaki, Mitsuko Iguchi, Kie Nakatani, Eri Sasabe, Yukio Yoshioka, Tetsuya Yamamoto, Kenji Yamagata

**Affiliations:** 1 Department of Oral and Maxillofacial Surgery, Kochi Medical School, Kochi University, 185-1, Kohasu, Oko-Cho, Nankoku-City 783-8505, Japan; nakatani_kie@kochi-u.ac.jp (K.N.); yoshieri@kochi-u.ac.jp (E.S.); k73347145@kochi-u.ac.jp (Y.Y.); y-kenji@kochi-u.ac.jp (K.Y.); 2 Kochi Institute for Core Sample Research, X-Star, Japan Agency for Marine-Earth Science Technology (JAMSTEC), B200 Monobe, Nankoku-City 783-8502, Japan; motoo@jamstec.go.jp; 3 Marine Core Research Institute, Kochi University, B200 Monobe, Nankoku-City 783-8502, Japan; jm-takuya@kochi-u.ac.jp; 4 Diagnostic Pathology Department, Kochi Medical School Hospital, 185-1, Kohasu, Oko-Cho, Nankoku-City 783-8505, Japan; iguchim@kochi-u.ac.jp; 5 YAMAKIN Next Generation Dental Medicine Development Course, Kochi Medical School, Kochi University, 185-1, Kohasu, Oko-Cho, Nankoku-City 783-8505, Japan; yamamott@kochi-u.ac.jp

**Keywords:** phosphoglyceride crystal deposition disease, mandible, recurrence, malignant tumor, positron emission tomography-computed tomography, Raman spectroscopy, literature review

## Abstract

**Background and Clinical Significance**: Phosphoglyceride crystal deposition disease (PCDD) is an extremely rare condition characterized by the deposition of phosphoglyceride crystals, occasionally forming tumor like lesions that present significant diagnostic challenges. Here, we report, to our knowledge, the first documented recurrent case of PCDD confined to the mandible, which clinically and radiologically mimicked a malignant bone tumor. **Case Presentation**: An 80-year-old female patient presented with a progressively enlarging mandibular mass, and imaging studies demonstrated an osteolytic lesion with cortical bone destruction and marked fluorodeoxyglucose uptake on positron emission tomography-computed tomography, raising a strong suspicion of malignancy. Histopathological examination revealed foreign-body granulomatous inflammation with characteristic crystal deposition, and the diagnosis of PCDD was definitively established through the combined use of gold hydroxamic acid staining, Raman spectroscopy, and ultrastructural analysis. Although surgical excision with curettage was initially achieved, local recurrence was observed 6 years later, indicating the potential for long-term disease persistence. In addition, a comprehensive literature review conducted in accordance with the PRISMA guidelines was performed to summarize previously reported cases of PCDD, with particular attention to anatomical distribution, radiological characteristics, recurrence patterns, and proposed pathogenic mechanisms. The review confirmed the extreme rarity of mandibular involvement and demonstrated that recurrence can occur apparently even after surgical treatment. **Conclusions**: This case underscores the importance of a multimodal diagnostic strategy integrating imaging, histopathology, and spectroscopic analyses for the accurate identification of PCDD and highlights the necessity of histopathological confirmation to prevent unnecessary aggressive treatment.

## 1. Introduction

Phosphoglyceride crystal deposition disease (PCDD) is an exceedingly rare clinicopathological entity characterized by the accumulation of phosphoglyceride (PG) crystals—lipid components normally present in cellular and organelle membranes—within soft tissues or bones, particularly in non-articular regions, accompanied by granulomatous inflammation composed of macrophages and multinucleated giant cells [1,2]. These crystals can induce foreign-body granulomatous inflammation. Unlike well-established crystal deposition diseases such as gout or pseudogout, which primarily affect joints, PCDD is distinguished by granulomatous lesions that arise at sites of prior surgical procedures, repeated injections, or traumatic stimuli [3,4,5].

Histopathologically, PCDD is characterized by asteroid- or string-like birefringent crystals under polarized light and positive staining with the gold hydroxamic acid (GHA) method [4,5]. The PG nature of these crystals has been confirmed using Fourier transform infrared (FTIR) spectroscopy, X–ray microanalysis, microsampling mass spectrometry, and Raman spectroscopy [4,5,6,7]. Although the precise pathogenesis remains unclear, localized tissue injury with subsequent abnormal lipid metabolism has been proposed as a potential mechanism [8]. Most affected individuals are otherwise healthy adults, and the disease generally follows an indolent clinical course.

Despite its benign nature, PCDD often presents as a mass-forming lesion with aggressive radiological features, including high fluorodeoxyglucose (FDG) uptake on positron emission tomography-computed tomography (PET-CT), and is therefore frequently misdiagnosed as malignant tumor [9,10]. To date, only a limited number of cases have been reported worldwide, with mandibular involvement being particularly rare. Recurrence has been infrequently documented, and its clinical characteristics remain poorly understood.

Here, we report a rare recurrent case of PCDD confined to the mandible that radiologically mimicked a malignant bone tumor, together with a review of the literature to clarify its clinicopathological features and recurrence patterns.

## 2. Materials and Methods

### 2.1. Literature Search Strategy

**Figure 1:**
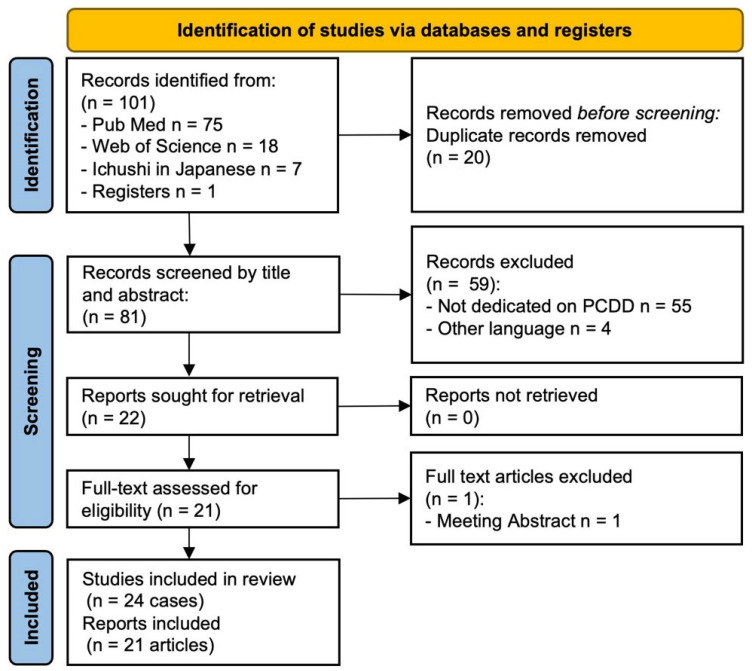
PRISMA flow diagram of the literature search and study selection process.

A literature review was conducted in accordance with the PRISMA guidelines [11]. Relevant articles were identified through database searches and the manual screening of reference lists to collect previously reported cases of PCDD. The review aimed to summarize clinical characteristics, anatomical distribution, radiological findings, treatment modalities, and recurrence patterns. As this review was intended to provide an overview of reported cases, it was not designed as a systematic review or meta-analysis. A literature search was conducted in PubMed, Web of Science, and the Ichushi (Japan Medical Abstracts Society) databases, covering studies published in English and Japanese from 1992, when this entity was first described by Kubo et al. [1], through August 2025, and the original landmark report by Kubo et al. (1992), which established the concept of phosphoglyceride crystal deposition disease, was specifically reviewed and cited to provide historical and conceptual context. The search utilized free-text terms in the “All fields” section. The question was as follows: (Phosphoglyceride crystal deposition) OR (Phosphoglyceride crystal deposition disease) OR (PCDD) OR (PGDD). In addition to the search, one well-recognized landmark report was added manually to ensure comprehensive coverage. The study selection process is illustrated in the PRISMA flow diagram (Figure 1).

### 2.2. Specimen Preparation and Acid/Alkali Exposure

Formalin-fixed, paraffin-embedded sections containing crystalline material were treated with either 30% acetic acid or 0.1 N NaOH to assess solubility and potential gas evolution [4,5,8].

### 2.3. Detection of PG by the GHA Method

PGs were detected on paraffin-embedded tissue sections using the gold hydroxamic acid (GHA) method. Formalin-fixed, paraffin-embedded tissue blocks were sectioned at a thickness of 5 μm. The sections were deparaffinized and rinsed three times with distilled water. Hydrolysis was performed by incubating the sections in an alkaline hydroxylamine solution (20 mL of 5% hydroxylamine hydrochloride mixed with 20 mL of 12% sodium hydroxide) for 20 min at room temperature. The sections were washed again in distilled water (three times for 5 min each, total 15 min) and then incubated for 2–4 h in silver solution (0.2 g of NH_4_NO_3_, 0.1 g of AgNO_3_, 1.0 g of 2.5% NaOH, and 99 mL of distilled water) under electric light or sunlight. The sections were subsequently washed three times in distilled water (10 min each), immersed in 1% acetic acid for 5 min, and washed again three times in distilled water (10 min each). The sections were then treated with 0.2% gold chloride solution for 10 min, rinsed in distilled water, and immersed in 5% sodium thiosulfate solution for 5 min. Finally, the sections were washed with distilled water, dehydrated through graded alcohols, cleared, and mounted [12,13].

### 2.4. Immunohistochemistry

Immunohistochemical labeling was performed using 4-μm-thick sections from formalin-fixed and paraffin-embedded tissues. Slides were deparaffinized and subjected to antigen retrieval by Pronase treatment. Staining was performed on the automated Ventana Benchmark ULTRA system using the biotin-free VENTANA ultraView Universal DAB Detection Kit (Ventana Medical Systems, Tucson, AZ, USA). A CD68 antibody was applied at indicated dilutions (clone PGM-1, mouse monoclonal, dilution 1:100; DAKO, Glostrup, Denmark). Appropriate positive and negative controls were included in the immunolabeling procedure.

### 2.5. Raman Spectroscopy

Cell samples and related standard material (lecithin) on glass plates were analyzed with a confocal Raman microscope (WITec Alpha 300R; WITec GmbH, Ulm, Germany) at the Kochi Institute for Core Sample Research, JAMSTEC. The instrument was equipped with a 532-nm laser and a ×50 objective lens (Carl Zeiss AG, Oberkochen, Germany), providing a spot size of approximately 0.9 µm (calculated from a numerical aperture of 0.75). The laser power was set to approximately 5 or 10 mW (measured at the objective lens) depending on the sample. Spectral data were collected over the range of 0–3700 cm^−1^ using a grating with 600 grooves/mm. For each analytical spot, spectra were accumulated for 100 s with a 1-s integration time and 100 scans. Prior to the measurements, the Raman shift was calibrated using a silicon wafer.

### 2.6. Transmission Electron Microscopy

The specimens were fixed with 2% glutaraldehyde in 0.1 M phosphate buffer (pH 7.3) for 2 h at 4 °C. They were then postfixed with 1% osmium tetroxide in 0.1 M phosphate buffer (pH 7.3) for 1 h at 4 °C and dehydrated in a graded series of ethanol. Following dehydration, the specimens were transferred to propylene oxide and embedded in Epon 812 (TAAB Laboratories Equipment Ltd., Aldermaston, Berkshire, UK). The specimens were observed using a JEM-1400Plus electron microscope (JEOL Ltd., Tokyo, Japan).

## 3. Case Presentation

**Figure 2:**
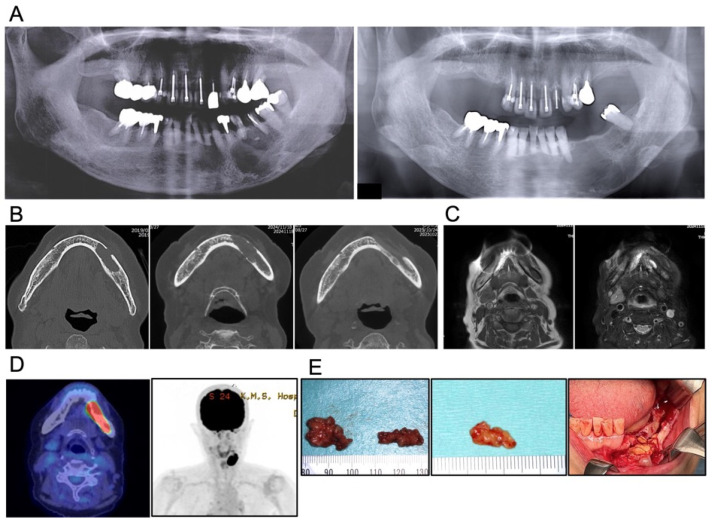
Radiological and macroscopic findings. (**A**) Panoramic radiographs obtained in 2019 (left) and in 2025 (right) show radiolucent lesions in the left mandibular molar region. (**B**) Cone-beam CT images from 2019 (left), 2025 before surgery (middle), and six months postoperatively (right). The recurrent lesion is larger than the initial lesion and demonstrates irregular bone resorption and loss of cortical bone continuity. Postoperative images show new bone formation without evidence of recurrence. (**C**) MRI findings in 2025. The lesion exhibits well-defined margins and is iso-intense relative to muscle on T1-weighted images (left) and heterogeneously hyperintensity on fat-saturated T2-weighted images (right). (**D**) PET-CT shows intense FDG uptake throughout the mandibular lesion, with no evidence of cervical or pulmonary metastasis. (**E**) Macroscopic appearance of the excised specimens in 2019 initial (left), the 2025 biopsy (middle), and the 2025 excision (right). All specimens demonstrate pale-yellow, adipose-like, soft tissue consistent with PCDD.

**Figure 3:**
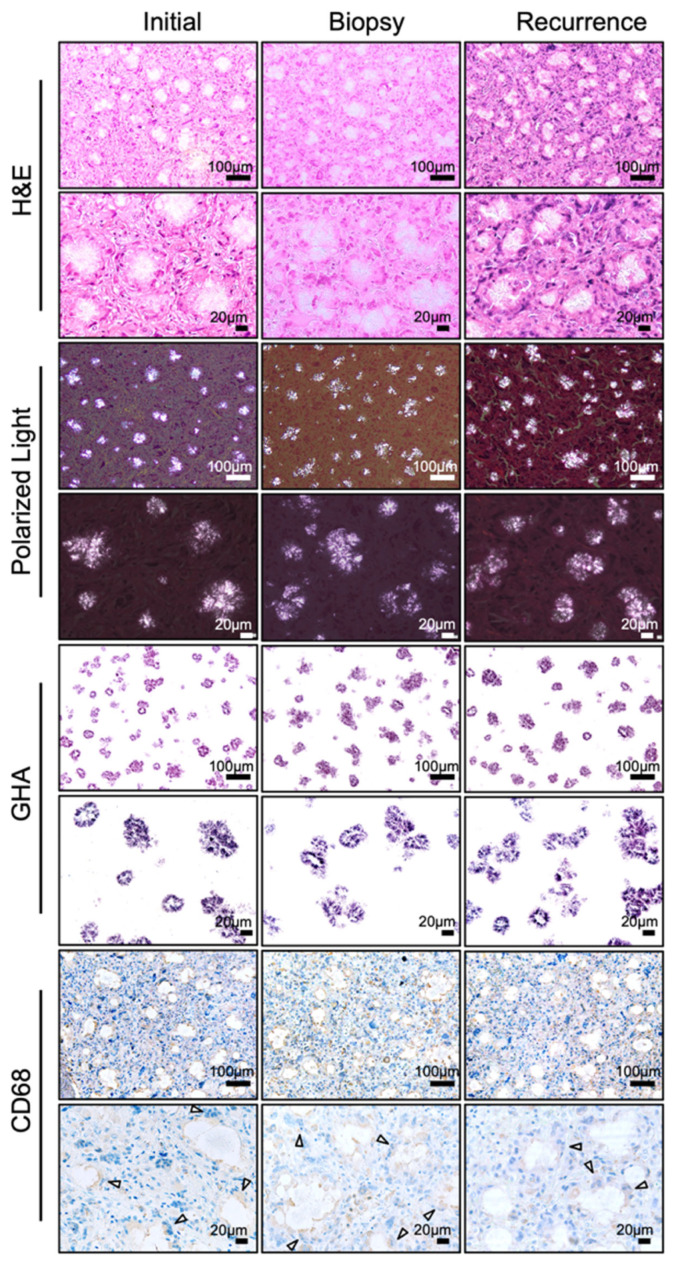
Histopathological findings. Representative sections from the initial, biopsy and recurrent specimens. From top to bottom: H&E staining, polarized light microscopy, GHA staining, and immunohistochemistry for CD68. Upper panels show low magnification (scale bar = 100 μm) and lower panels show high magnification (scale bar = 20 μm). H&E staining demonstrates foreign-body granulomas with crystalline deposits surrounded by multinucleated giant cells and macrophages. Under a polarized light, the crystals appear needle- or thread-like and exhibit birefringence. Crystals stain positively with the GHA method, which specifically detects PGs. CD68 immunostaining highlights macrophages and multinucleated giant cells surrounding the crystals, particularly at the plasma membrane facing the deposits (white arrowheads).

The patient was a 74-year-old Japanese woman who was referred to the Department of Oral and Maxillofacial Surgery at our university hospital in Japan in 2019 with pain in the left mandibular molar region and a gingiva fistula at the apical area of 35. No remarkable systemic symptoms were noted. She had a medical history of hypertension and hyperlipidemia, both of which were well-controlled with calcium channel blockers, angiotensin II receptor blockers (ARBs), and HMG-CoA reductase inhibitors. A panoramic radiograph revealed a cyst-like radiolucent area measuring 25 × 20 mm in the same region (Figure 2A, Left). Cone-beam computed tomography (CBCT) demonstrated a well-circumscribed unilocular radiolucent lesion involving the root apices of teeth 35, 36, and 37 (Figure 2B, Left). Because tooth 36 had undergone root canal treatment and there was no buccolingual swelling of the mandibular bone, the lesion was clinically diagnosed as a radicular cyst. Under general anesthesia, teeth 35, 36, and 37 were extracted, and the cystic lesion was enucleated. Contrary to the preoperative diagnosis, the excised tissue was solid and yellowish-white, resembling adipose tissue and was removed in two fragments measuring 20 × 20 mm and 20 × 10 mm, respectively (Figure 2E, Left). Although the lesion extended to the mandibular canal, the inferior alveolar neurovascular bundle was preserved as far as possible. Because the lesion was solid and its boundary with the surrounding normal tissue was ill-defined, thorough curettage and irrigation of the adjacent bone and soft tissue surfaces were performed, and the wound was managed as an open wound until uneventful epithelialization was achieved. The specimen was histopathologically diagnosed as a foreign-body granuloma. Polarized-light observation revealed numerous crystal deposits; however, no additional special examinations were performed at that time (Figure 3, left).

**Figure 4:**
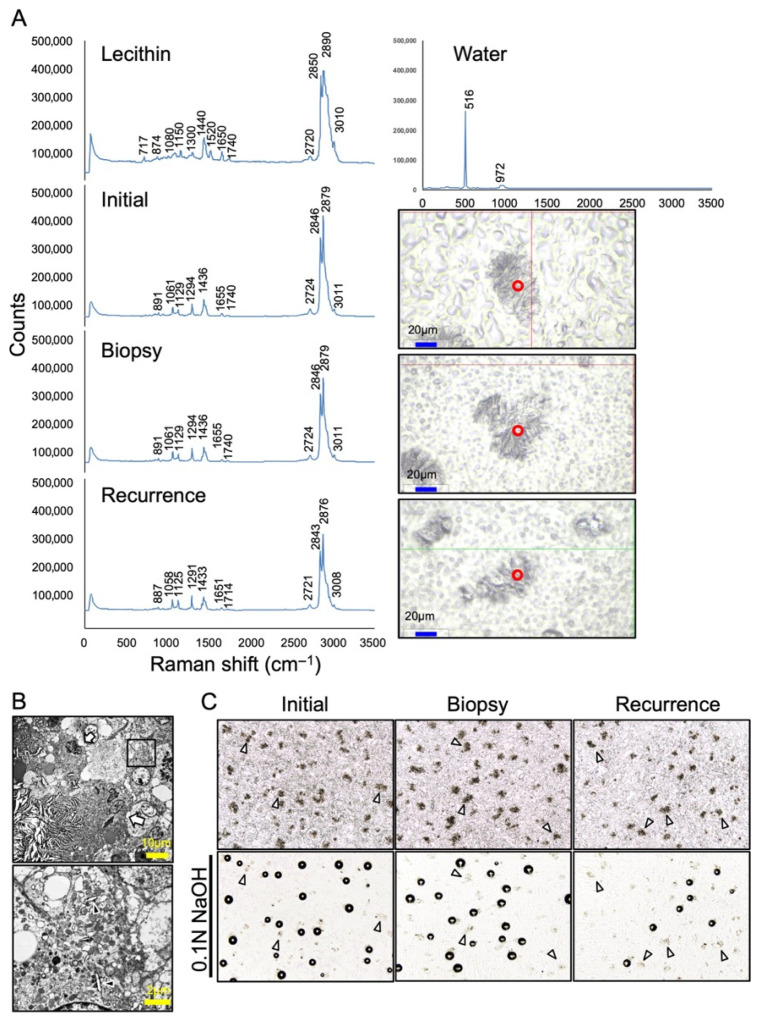
Spectroscopic and ultrastructural analysis of the crystalline deposits. (**A**) Raman spectroscopy of the specimen (initial, biopsy and recurrence) compared with control (lecithin and water). The analyzed regions are indicated by the red circle in right panels respectively. Peaks corresponding to PG functional groups—P–O–C vibrations (~890 cm^−1^), C=O stretching (1714 and 1740 cm^−1^), chain-derived signals (1290 and 1440 cm^−1^), CH_2_ bending/scissoring (1290–1445 cm^−1^), and unsaturated fatty acid signals (1651–1655 cm^−1^, cis C=C)—support the presence of PGs and are summarized in Table 1. (**B**) Transmission electron microscopy shows thread-like crystals surrounded by multinucleate giant cells (white arrows) in upper panel and in the lower panel (area boxed in black in the upper panel) crystalline material within the cytoplasm of macrophage (black arrowheads), suggesting phagocytosed residual bodies (original magnification upper × 500; lower × 3000). (**C**) Chemical reactivity of crystalline deposits. Paraffin-embedded sections were treated with 30% acetic acid or 0.1 N NaOH. Gas formation was observed after NaOH exposure, whereas no visible reaction occurred with acetic acid. Some crystal regions did not produce gas after NaOH treatment (white arrowheads).

Six years after the initial surgery, in January 2025, at the age of 80, the patient experienced pain again in the same region and visited a local dentist. A panoramic radiograph demonstrated a recurrent radiolucent lesion measuring 34 × 25 mm in the left mandible, and she was referred to our department with suspicion of a mandibular tumor (Figure 2A, Right). CBCT showed a radiolucent lesion larger than the initial one, with irregular bone resorption at the margins and partial loss of cortical bone continuity (Figure 2B, middle). Magnetic resonance imaging (MRI) revealed a well-demarcated lesion exhibiting iso-intensity relative to muscle on T1-weighted images and heterogeneous hyperintensity on fat-saturated T2-weighted images (Figure 2C). Positron emission tomography-computed tomography (PET-CT) demonstrated intense fluorodeoxyglucose (FDG) uptake in the lesion, with a maximum standardized uptake value (SUVmax) of 28.07, occupying more than one-third of the mandible. Notably, despite the intense and extensive FDG uptake within the mandibular lesion, no abnormal FDG accumulation was observed in the cervical lymph nodes or lungs (Figure 2D). Given these imaging findings, a malignant tumor was strongly suspected. To obtain a definitive diagnosis, a biopsy was performed under local anesthesia. The specimen consisted of yellowish-white, fat-like tissue resembling the previous surgical specimen. Histopathological evaluation again revealed foreign-body granuloma (Figure 2E, Middle). On the basis of these findings, recurrence of the granulomatous lesion was diagnosed, and the patient underwent tumor enucleation under general anesthesia. The resected specimen exhibited macroscopic features similar to those observed previously—pale yellow, firm, adipose-like tissue mildly adherent to the mandibular bone—and measured 32 × 15 mm (Figure 2E, Right). Hematoxylin and eosin (H&E) staining showed numerous needle-shaped crystalline structures surrounded by multinucleated giant cells and macrophages (Figure 3, Middle and Right). Polarized-light microscopy demonstrated asteroid- and string-like birefringent crystals. Gold hydroxamic acid (GHA) staining yielded strong positivity, confirming the presence of phosphoglycerides (PGs). Raman spectroscopy revealed spectral patterns nearly identical to those of phosphatidylcholine (lecithin) used as a control, verifying the diagnosis of phosphoglyceride crystal deposition disease (PCDD). Retrospective re-examination of the initial 2019 specimen demonstrated identical histological features, confirming that both lesions represented PCDD (Figure 3 and Figure 4A). At 6 months postoperatively, CBCT showed new bone formation around the surgical site, and no radiological or clinical evidence of recurrence was detected (Figure 2B, Right).

**Table 1:** Summary of Raman spectroscopic features of phosphoglyceride crystal deposits.

Vibration	Typical Range (cm^−1^)	Observed in S1–S3
P–O–C (phosphate–glycerol)	840–900	887–891
PO_2_^−^ symmetric stretch	1080–1095	1080–1129
Ester C=O	1710–1745	1714/1740
C=C (cis)	1648–1660	1651–1655
CH_2_ bending	1290–1300	1290
CH_2_ scissoring	1430–1445	1440
C–H stretching	2850–2950	–
=C–H stretching	~3005–3020	–
Water δ(H_2_O)	~1640	–
Water ν(O–H)	3100–3650 (broad)	–

## 4. Discussion

### 4.1. Epidemiological and Clinical Features

PCDD is an exceptionally rare disease entity, and its pathogenesis remains incompletely understood. The present case is notable for its occurrence in the mandible and for local recurrence after surgical treatment, features that have been rarely documented in the literature. To our knowledge, this represents the first reported recurrent case of PCDD confined to the mandible.

**Figure 5:**
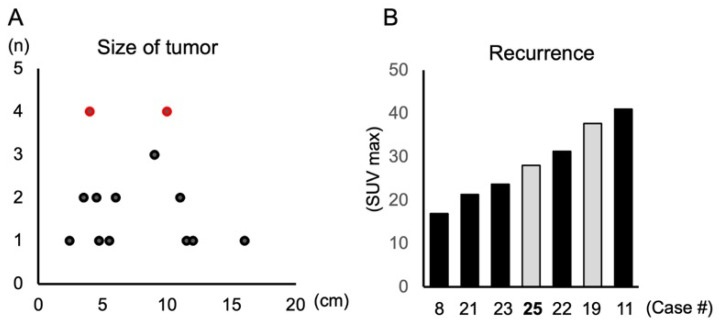
Clinicopathological distribution and recurrence analysis. (**A**) Distribution of maximum tumor size among all 25 reported PCDD cases showed bimodal peaks which were highlighted red dots at approximately 4 cm and 10 cm. (**B**) Relationship between preoperative FDG-PET SUVmax and recurrence in surgically treated cases. Cases with SUVmax > 28, including Case 19 and the present Case 25, demonstrated recurrence which highlighted by gray bars, suggesting an association between high metabolic activity and recurrence risk.

Twenty-one publications comprising 24 cases were identified, and together with the present case, a total of 25 cases were included for analysis. The clinical characteristics of all cases, including the present case, are summarized in Table 2, and their anatomical distribution and clinical course are presented in Table 3. Five additional reports published only in Japanese were excluded from the main analysis. This may reflect reporting bias rather than true epidemiological distribution. Indeed, 24 out of 25 reported cases originated from Japan, although the reason for this geographic clustering remains uncertain. The mean age of affected patients was 63 ± 13 years, with a female predominance (female: male = 1.8:1), consistent with prior summaries [14,15]. One possible explanation is the higher prevalence of surgical histories unique to women, such as cesarean section. Approximately 30% of lesions occurred in bone, while the remainder were in soft tissues. Lesions most frequently involved the abdominal wall and soft tissue region (*n* = 7), followed by the mediastinum and ventricular apical (*n* = 4). Surgical procedures such as cesarean section, appendectomy, and cardiac septal defect repair were commonly documented antecedents, suggesting that prior tissue injury may act as a local trigger for phosphoglyceride crystallization [16,17]. To date, only two cases had been reported in the head and neck region [4,15]. Most lesions were asymptomatic and often discovered incidentally on imaging examination during follow-up. Although usually solitary, approximately 30% of cases exhibited multiple or systemic involvement. The mean tumor diameter was 7.3 ± 3.4 cm, showing a tendency toward larger size in males (8.3 ± 3.1 cm) than females (7.4 ± 3.6 cm), in those over 65 years (under 64 years old [7.0 ± 4.2 cm] and over 65 years old [8.0 ± 2.9 cm]), and in lesions originating from soft tissue (soft tissue [8.14 ± 3.4 cm] and hard tissue [6.22 ± 3.0 cm]), although statistical significance was not achieved due to the limited number of cases. Size distribution appeared bimodal, with peaks near 4 cm and 10 cm (Figure 5A). Reported growth rates vary; while some lesions enlarge slowly, others remain unchanged for years [18,19,20]. Notably, clinical symptoms are not uncommon in PCDD. Although the disease is often described as asymptomatic, approximately half of the reported cases, including the present case, showed clinical manifestations such as pain or local discomfort. Superficially located lesions are often recognized as mass enlargement or swelling [15,21], whereas deeply located lesions may cause nonspecific symptoms due to the compression of adjacent tissues or vascular structures [18,19,20]. Pain has been reported in several cases [7,10,14,17,22,23,24], and its presence does not exclude the diagnosis of PCDD. Rather, pain may reflect secondary effects of lesion enlargement, such as compression of the surrounding tissues or the involvement of adjacent structures, and may provide a clinical clue to lesion location.

### 4.2. Radiological Characteristics and Diagnostic Challenges

Radiologically, PCDD often presents as a well-demarcated yet heterogeneous mass that may exhibit calcification or necrosis [9,15,24]. On MRI, the lesions are typically iso- to hypointense on T1-weighted images and heterogeneously hyperintense on T2-weighted sequences [22]. A striking feature is the remarkable FDG uptake on PET-CT; among 10 cases for which data were available, the mean SUVmax was 29.1 ± 8.5 (Table 2). Such intense uptake has frequently led to misdiagnosis as malignancy; indeed, 16 out of 19 cases were clinically suspected to represent malignant tumors (Table 2). In our case, although the initial demonstrated a relatively clear margins on CT, the recurrence showed cortical bone destruction and irregular margins, hypo-intensity on T1-weighted and hyper-intensity on T2-weighted MRI, and an SUVmax of 28.07 on PET-CT, all findings that strongly suggest malignancy of the mandible [27,28]. Without prior biopsy, segmental mandibulectomy might have been performed unnecessarily. Based on the clinical and radiological findings, the differential diagnoses included medication-related osteonecrosis of the jaw (MRONJ), chronic osteomyelitis, primary intraosseous carcinoma of the mandible, and metastatic mandibular lesions. MRONJ and chronic osteomyelitis were considered but deemed unlikely due to the absence of antiresorptive or antiangiogenic agent use, exposed bone, peripheral sclerosis, mixed osteolytic–osteoblastic changes, and supportive laboratory or clinical findings. In contrast, primary intraosseous carcinoma and metastatic lesions could not be excluded preoperatively. Despite intense FDG uptake on PET imaging, the absence of cervical lymph node metastasis or abnormal FDG accumulation in other organs did not rule out malignancy, as metastasis from organs with physiological FDG uptake and the limited nodal spread characteristic of primary intraosseous carcinoma due to the periosteal barrier remained possible. Accordingly, histopathological examination was required to establish a definitive diagnosis. Therefore, histopathological confirmation is essential before undertaking extensive surgical resection.

### 4.3. Histopathological and Immunohistochemical Features

PCDD typically demonstrates radially arranged needle- or thread-like crystals surrounded by CD68 positive macrophages and multinucleated giant cells, forming a foreign-body granuloma [5,6,9,15,18,21,23,29]. CD68 is a glycoprotein localized in the lysosomes and endosomes of macrophages and monocytes. Interestingly, CD68 was strongly positive in multinucleated giant cells, particularly at the plasma membrane facing the crystals, as demonstrated by immunohistochemical staining (Figure 3). This pattern suggests active phagocytosis and attempted degradation of the deposited material. Birefringence under polarized light and specific positivity with GHA staining, which visualizes the localization of phospholipids, are diagnostic hallmarks [26]. In fact, GHA staining has been performed as the basis for diagnosing PCDD in 12 out of the 24 reported cases (Table 1). The present case also reproduced these features across all specimens, including the initial, biopsy, and recurrent samples. Previous studies using electron microscopy have shown lysosome-like organelles containing crystalline inclusions within macrophages [8]. Consistent with these findings, multinucleated giant cells surrounding the crystals and crystal deposition within the cytoplasm of macrophages were also observed in the present case (Figure 4B).

### 4.4. Spectroscopic and Chemical Characteristics

The identity of phosphoglycerides (PGs) has been established through using FTIR spectroscopy, X-ray microanalysis, microsampling mass spectrometry, and Raman spectroscopy [4,5,6,7]. In the present case, Raman spectroscopy revealed characteristic peaks of glycerophospholipids—P–O–C vibration (~890 cm^−1^), ester C=O stretching (1714, 1740 cm^−1^), CH_2_ bending/scissoring (1290, 1440 cm^−1^) and unsaturated C=C stretching (1651–1655 cm^−1^)—confirming the PG composition (Figure 4A and Table 3). These findings indicate the fundamental structure of glycerophospholipids, consisting of a glycerol backbone, two fatty acids, and a phosphate group. Interestingly, while phosphatidylcholine (lecithin) showed a strong Raman band at 717 cm^−1^, our specimens exhibited minor peaks at 734, 782, 826, and 848 cm^−1^, indicating that the deposited crystals may comprise a mixture of PG species rather than a single lipid. Because headgroup-specific Raman signals are often weak, complementary analyses such as FTIR spectroscopy or microsampling mass spectrometry are required for precise compositional analysis. When paraffin sections containing crystals were treated with 0.1 N NaOH, bubble formation was selectively observed, consistent with earlier reports, whereas 30% acetic acid caused no visible reaction [4,5,8]. Because hydrolysis of PGs by NaOH or acetic acid should not normally produce gas, these bubbles may represent a secondary chemical reaction with other components rather than PG hydrolysis itself [30]. Not all crystal deposits produced bubbles, further suggesting compositional heterogeneity (Figure 4C).

### 4.5. Pathogenesis

A typical cellular membrane is composed of a lipid bilayer, and the distribution of its constituent phospholipid molecules is not uniform between the outer and inner layers. PGs are major components of cell and organelle membranes, with phosphatidylcholine predominating in the outer layer, whereas phosphatidylethanolamine, phosphatidylserine, and phosphatidylinositol are mainly localized to the inner layer. Among these, phosphatidylcholine (formerly lecithin) is the most abundant [31,32]. Under normal physiological conditions, these lipids are continuously degraded by hydrolysis into fatty acids and glycerol by phospholipase A_2_ in macrophage lysosomes [33]. When excessive cellular destruction occurs—such as after trauma, injection, or surgery—large amounts of PGs may be released into the extracellular space. The resulting overload can impair lysosomal degradation, leading to crystallization. Other potential contributing factors include chronic steroid use, use of chloroquine-class antimalarials or antiarrhythmic drugs such as amiodarone, and exposure of inorganic agents such as silica or asbestos, all of which may inhibit lysosomal enzymatic activity [34]. However, no consistent associations have been identified across reported cases. In the present case, chronic irritation from long-term dental treatment may have acted as a local trigger, although a single causal factor could not be definitively determined. The possible involvement of calcium within PG crystals as a counterion has been suggested in previous studies [1,4,5,7], although von Kossa staining was negative in our specimen. Calcium incorporation may inhibit PG-degrading enzymes, thereby perpetuating crystal accumulation and chronic granulomatous inflammation.

### 4.6. Different Diagnoses: Comparison with Other Crystal-Induced Disorders

Several other diseases involve crystal deposition, including gout (uric acid), pseudogout (calcium pyrophosphate), basic calcium phosphate (BCP) crystal deposition disease, kidney or urinary stones with calcium crystals, and hydroxyapatite-related calcific tendinitis [35,36,37]. Among these, hydroxyapatite deposition disease shows pathogenetic similarity to PCDD, in that local mechanical or hypoxic injury induces mineral precipitation and chronic inflammation. In contrast, metabolic dysregulation underlies the pathogenesis of gout, pseudogout, and BCP disease. Thus, PCDD likely represents a distinct pathological response to local tissue injury rather than a systemic metabolic disorder. In the present case, no additional investigations were performed to screen for other asymptomatic crystal deposition lesions, as the diagnosis of PCDD was established only after excision of the recurrent lesion and subsequent specialized examinations. However, gout was clinically excluded based on the absence of characteristic symptoms and perioperative laboratory findings.

### 4.7. Prognosis and Recurrence

Recurrence was documented in 4 out of 15 surgically treated cases (26.7%), all occurring in women (Table 1 and Table 2), suggesting a possible sex-related factor [4,6,25]. Nevertheless, given the indolent and slowly progressive feature of PCDD, the possibility of regrowth from residual disease cannot be completely excluded. Although neither tumor size nor age predicted recurrence, a high SUVmax (>28) on preoperative PET-CT appears to be associated with a higher probability of recurrence (Figure 5B). However, this observation is based on a very limited number of cases and should be interpreted with caution. This finding should therefore be regarded as exploratory and hypothesis-generating rather than predictive. The present case recurred six years after the initial surgery, despite apparent surgical treatment, reinforcing the need for long-term observation. At present, the patient remains disease-free six months post-surgery (Figure 2B, right). It should be noted that the postoperative follow-up period in the present case was limited to six months, which represents short-term follow-up, and a longer observation is required to fully assess the risk of recurrence.

## 5. Conclusions

PCDD is an exceptionally rare condition characterized by ectopic crystallization of PGs, which are normally integral components of cell and organelle membranes. Local tissue injury or inflammatory stimuli may disturb phospholipid metabolism and lysosomal degradation within macrophages, leading to crystal accumulation and granulomatous inflammation.

Clinically and radiologically, PCDD often mimics malignancy because of its mass-forming behavior, bone destruction, and intense FDG uptake on PET-CT. Therefore, definitive diagnosis relies on histopathological confirmation, including birefringence under polarized light, positive GHA staining, and spectroscopic identification of PGs. Awareness of these features is crucial to prevent unnecessary aggressive surgical interventions.

The present case represents the first documented recurrence of PCDD confined to the mandible, with recurrence occurring six years after initial surgical treatment. The recurrent lesion exhibited histopathological and spectroscopic findings identical to those of the primary lesion, confirming disease recurrence. This case further demonstrates that even minimally invasive dental procedures, such as endodontic therapy, may act as triggering factors and that differentiation from primary intraosseous carcinoma or tumor recurrence can be extremely challenging without histological evaluation.

Our review of 25 cases revealed a female predominance, frequent association with prior local interventions, and a recurrence rate of approximately 30%. Although lesions showing high SUVmax values on PET-CT tended to be associated with recurrence, this observation is based on a limited number of cases and should be interpreted with caution. Accordingly, this finding should be regarded as exploratory and hypothesis-generating rather than predictive. Despite the limited number of reported cases, current evidence supports the concept that PCDD represents a localized metabolic disorder rather than a systemic lipid abnormality. Continued accumulation of well-characterized cases is essential to clarify its pathogenesis, identify risk factors for recurrence, and establish optimal diagnostic and management strategies.

Furthermore, although no cases of PCDD arising in postoperative sites of malignant tumors have been reported to date, differentiation from true tumor recurrence would be extremely challenging should such a scenario occur. Consequently, even when a recurrent malignancy is suspected, histopathological evaluation remains indispensable for establishing an accurate diagnosis when clinical findings are inconclusive.

These observations suggest a potential association between metabolic activity and disease persistence.

## Figures and Tables

**Table 2:** Clinical characteristics of reported cases of PCDD.

No	Author	Country	Age	Sex	History	Location	Tissue	Maximum Tumor Size (cm)	Number of Tumors	PET-CT (SUV Max)	Suspicious for	Biopsy	GHA Stain	Recurrence
1	Kubo et al. 1992 [1]	Japan	58	M	Intramuscular injection	Buttock muscle	Soft	11	Single	No	NS	No (excisional biopsy)	NS	NS
2	Miura et al. 2000 [4]	Japan	62	F	Appendectomy, Periodontal surgery	Maxillary soft tissue Abdominal soft legion	Soft	11	Multiple (systemic)	No	NS	NS	Yes	Yes
3	Yachida et al. 2002 [8]	Japan	51	M	Appendectomy, Distal partial gastrectomy	Abdominal soft region	Soft	3.5	Multiple	No	Malignant lymphoid tumor	Yes	No	Follow
4	Miura et al. 2004 [5]	Japan	64	F	ND	Scapular	Hard	10	Single	No	NS	NS	Yes	NS
5		Japan	64	F	Gastrectomy	Abdominal soft region	Soft	4	Single	No	NS	NS	Yes	NS
6		Japan	58	M	Gastrectomy	Abdominal soft region	Soft	12	Single	No	NS	NS	Yes	NS
7	Nishimura et al. 2005 [22]	Japan	76	F	Appendectomy, Lumber anesthesia	Spine	Hard	NS	Multiple	No	NS	No (excisional biopsy)	No	NS
8	Shoji et al. 2007 [9]	Japan	37	M	Repair for a ventricular septal defect	Anterior mediastinum	Soft	6	Single	16.9	Malignant mediastinal (cardiac or thymic origin) tumor	Yes	No	No
9	Yamada et al. 2015 [25]	Japan	50	F	Cesarean delivery	Pelvic soft tissues	Soft	10	Multiple	No	Malignant ovarian tumor	No (excisional biopsy)	Yes	Yes
10	Tokue et al. 2018 [18]	Japan	45	F	Repair for atrial septal defect	Anterior mediastinum	Soft	9	Multiple	26	Malignant mediastinal tumor	Yes	No	Follow
11	Nakahara et al. 2018 [23]	Japan	57	M	Appendectomy	Abdominal wall	Soft	10	Single	41	Malignant soft tissue tumor (lymphoma, undifferentiated sarcoma or liposarcoma)	No (excisional biopsy)	No	No
12	Sato et al. 2020 [16]	Japan	42	M	Repair for atrial septal defect	Ventricular apical	Soft	4.5	Single	42.997	Malignant cardiac tumor	Yes	No	Follow
13	Ishida et al. 2021 [19]	Japan	85	F	Appendectomy, pacemaker implantation	Ovary	Soft	16	Single	No	Malignant ovarian tumor	No (excisional biopsy)	Yes	No
14		Japan	59	F	Repair for patent ductus arteriosus, caesarean delivery	Fourth rib bone	Hard	4	Single	21.8	Malignant bone tumor	Yes	Yes	Follow
15	Nakamura et al. 2021 [26]	Japan	84	F	Hysterectomy for uterine leiomyoma	Bilateral adnexa and pelvic wall	Soft	4.5	Multiple	No	Malignant soft tissue tumor	No (excisional biopsy)	Yes	No
16	Miki et al. 2021 [17]	Japan	48	F	Repair for a ventricular septal defect	Anterior mediastinum	Soft	9	Multiple	No	PCDD	Yes	No	No
17	Omar et al. 2022 [21]	Malaysia	69	M	Traumatic laceration	Calf	Soft	11.5	Single	No	Malignant soft tissue tumor	No (excisional biopsy)	No	No
18	Takeuchi et al. 2022 [20]	Japan	72	F	Atrial septum closure and tricuspid valvuloplasty	Atrium	Soft	4	Single	NS	Malignant cardiac tumor	Yes	Yes	Follow
19	Ohkura et al. 2022 [6]	Japan	60	F	Splenectomy	Abdominal soft region	Soft	5.5	Single	37.71	Malignant soft tissue tumor	Yes	No	Yes
20	Tsutsumi et al. 2023 [7]	Japan	71	M	Ileocecal resection	Intestine	Soft	6	Single	No	Submucosal tumor	Yes	Yes	No
21	Hori et al. 2023 [14]	Japan	76	F	Appendectomy	Scapular	Hard	4.7	Single	21.35	Malignant bone tumor	Yes	Yes	No
22	Furuzono et al. 2024 [24]	Japan	50	F	Cesarean delivery	Abdominal wall	Soft	9	Single	31.28	Malignant ovarian tumor	Yes	No	No
23	Matsutani et al. 2025 [10]	Japan	82	M	Ileocecal resection	Humerus	Hard	10	Single	23.7	Malignant bone tumor or chondrosarcoma	Yes	No	No
24	Saitou et al. 2025 [15]	Japan	81	F	tooth extraction	Maxilla	Hard	2.4	Single	No	Maxillary tumor	Yes	Yes	No
25	Present case	Japan	73	F	Dental treatment	Mandibular	Hard	4	Single	28.07	Malignant mandibular bone tumor	Yes	Yes	Yes

NS: Not shown, and SUV Max = Maximum standardized uptake value.

**Table 3:** Distribution of clinical and pathological features of PCDD.

Total Number of Cases *n* = 25 (Include Present Case)		
		Number of Cases
Distribution	Age	62.96 ± 13.4
	Sex (Male:Female)	9:16
	Tissue (Soft:Bone)	18:7
	Number (Single:Multiple)	18:7
	Size (cm) (*n* = 24)	7.37 ± 3.42
	SUV Max (*n* = 10)	29.08 ± 8.46
Clinical diagnosis	Malignant tumor	16
	PCDD	1
	Tumor	2
	NS	6
Treatment	Excision after biopsy	9
	Follow-up conservatively after biopsy	5
	Excisional biopsy	7
	NS	4
Prognosis	Disease Free	11
	Recurrence	4
	Follow-up conservatively	5
	NS	5

## Data Availability

The data presented in the study may be available from the corresponding author upon reasonable request.
